# Nonlinear relationship between serum Klotho and chronic kidney disease in US adults with metabolic syndrome

**DOI:** 10.3389/fendo.2024.1409560

**Published:** 2024-12-24

**Authors:** Xiaobin Lin, Lin Yang

**Affiliations:** National Clinical Research Center for Metabolic Diseases, Key Laboratory of Diabetes Immunology (Central South University), Ministry of Education, and Department of Metabolism and Endocrinology, The Second Xiangya Hospital of Central South University, Changsha, China

**Keywords:** α-Klotho, chronic kidney disease, metabolic syndrome, NHANES, L-shaped

## Abstract

**Background:**

Current evidence regarding the effects of serum Klotho among patients with metabolic syndrome (MetS) is scarce. This study explored the relationship between serum Klotho levels and the odds of chronic kidney disease (CKD) in middle-aged and older populations with MetS.

**Materials and methods:**

This cross-sectional study analyzed data from 4870 adults aged 40–79 years who participated in the National Health and Nutrition Survey (NHANES) from 2007 to 2016. CKD was identified at urinary albumin to creatinine ratio (UACR) of 30 mg/g or higher and/or an estimated glomerular filtration rate (eGFR) below 60 mL/min/1.73 m^2^. Measurement of serum Klotho concentration was determined via enzyme-linked immunosorbent assay (ELISA) and subsequently divided into four quartiles (Q1-Q4). The NHANES criteria were followed in calculating the sampling weights. Multivariable logistic regression models were employed to assess the correlation between Klotho and CKD, while generalized linear models with cubic spline functions and smooth curve fitting were utilized to detect any nonlinear relationship. Additionally, subgroup analysis and a range of sensitivity analyzes were conducted.

**Results:**

Results showed that a nonlinear L-shaped relationship existed between serum Klotho levels and CKD risk, with the lowest prevalence observed at 9.63–9.94 pg/mL Klotho concentrations. With a two-segment linear regression model, an inflection point of 9.88 pg/mL was noted. Hypertension status was identified as an interaction mediator (*P*_interaction_ = 0.006). Sensitivity analysis showed stable results.

**Conclusions:**

A nonlinear L-shaped relationship exists between serum Klotho levels and risks of CKD among middle-aged and older adults with MetS, with the lowest prevalence observed at 9.63 to 9.94 pg/mL Klotho concentrations. Our findings, if replicated, underscore the need to estimate the optimal serum Klotho concentrations and the consequential inverse relationship, thus implying the potential of Klotho as both a serum biomarker and a possible preventive or therapeutic intervention.

## Graphical Abstract

Created with BioRender.com.

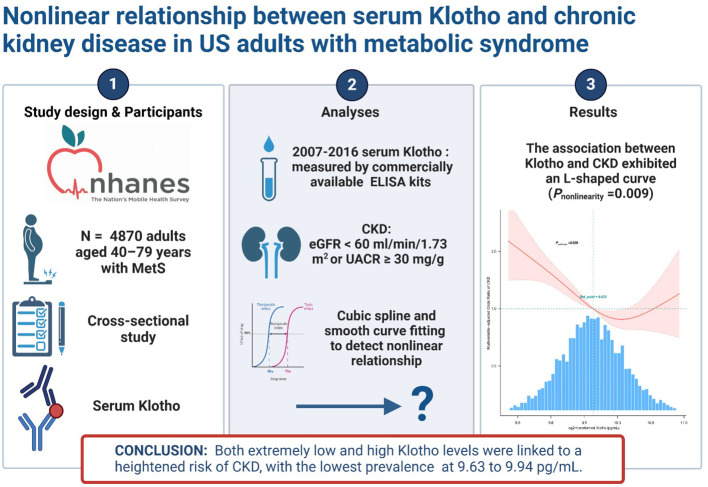

## 1 Introduction

Chronic kidney disease (CKD) poses a significant healthcare burden, with its insidious onset often leading to a lack of awareness, as evidenced by its high prevalence. In the United States, the prevalence of CKD in adults had been relatively stable at just under 15% for the last 15 years, possibly due to the increasing population of older individuals and high prevalence of diabetes, hypertension, obesity, cardiovascular disease, and other conditions (1, 2). CKD can progress to end-stage renal disease (ESRD), followed by complications such as cardiovascular disease, leading to increased infection and mortality.

Metabolic syndrome (MetS) represents a cluster of metabolic dysregulations that include hypertension, hyperlipidemia, central obesity, insulin resistance, hyperglycemia, and diabetes (3). MetS is intricately linked to the development, progression, and adverse outcomes of CKD, including poor renal and cardiovascular prognoses. MetS induces renal injury through oxidative stress, inflammation, hemodynamic changes, lipotoxicity, and insulin resistance (4, 5). These interconnected mechanisms amplify kidney damage and cardiovascular risks, including glomerular hypertrophy, tubulointerstitial fibrosis, microvascular remodeling, and podocyte damage (6). MetS increases CKD risk by 1.82-fold (7, 8), with 34.8-58.4% of CKD patients having MetS (9). CKD patients with concomitant MetS face higher risks of end-stage renal disease, mortality, and cardiovascular events (5, 6). Despite this clear association, there are no specific guidelines for managing kidney risk in MetS patients, underscoring the urgent need for further research to improve early prevention and outcomes in MetS patients with renal complications. MetS causes renal damage through mechanisms like oxidative stress, chronic inflammation, perirenal adipose tissue (PRAT) compression, and the overactivation of the renin-angiotensin-aldosterone system (RAAS), mineralocorticoid receptors (MR), and sympathetic nervous system (SNS), leading to renal hemodynamic changes, systemic insulin resistance, and lipotoxicity, which ultimately result in kidney damage and cardiovascular diseases (5). In obese patients, visceral adipose tissue exhibits elevated levels of angiotensinogen, increased leptin secretion, and upregulated MR expression also contribute to the development and progression of hypertension (6, 10). These changes collectively cause imbalances in arterial dilation and constriction, increase intrarenal pressure, and lead to glomerular damage, thereby elevating the risk of CKD (5). CKD and MetS are closely linked to several age-related diseases like cardiovascular disease, diabetes, and cancer. CKD significantly elevates cardiovascular risk, while MetS is a crucial predictor of type 2 diabetes and cardiovascular events (6, 10). Studies indicate that CKD and MetS can increase cancer risks through chronic inflammation, oxidative stress, and metabolic dysregulation (11). These conditions often coexist in elderly populations, creating complex multimorbidity that challenges clinical management. Hence, understanding their interconnections is crucial for developing effective prevention and treatment strategies for the aging population. Therefore, it is essential to identify additional effective biomarkers that could be detected in the early stages of CKD to indicate its course, thus tracking the disease’s progression and preventing unfavorable outcomes.

Klotho, also known as α-Klotho, was fortuitously identified in 1997 by Kuro-o et al. (12) as a gene associated with decreased life expectancy. Transmembrane protein Klotho is primarily expressed in the distal convoluted tubules (13). The extracellular domain of Klotho is released into the bloodstream after being cleaved by a protease or by alternative splicing. This circulating Klotho functions as a hormone that exerts pleiotropic effects including antioxidative stress, antisenescence, and antiapoptotic effects (14). Klotho is pertinent to various age-related disorders, including cardiovascular disease (15), chronic kidney disease (16),diabetes (17, 18), and cancer (19).

In recent years, there has been extensive research on the relationship between soluble Klotho and renal function in patients with CKD. A reno-protective activity of Klotho has been reported in various animal models of acute kidney injury (AKI) (20)and CKD (21, 22). To date, Klotho levels (serum and urine) have been positively correlated to eGFR in adults with CKD (23). Restoration of Klotho attenuates vascular calcification associated with CKD (24). Of note, the associations between Klotho and CKD risk among general populations are controversial. A cohort study involving patients with CKD (stages 1–5) revealed an independent relationship between serum Klotho levels and the combined outcome of doubling serum creatinine, kidney failure, or death after adjusting for age, blood pressure, eGFR, diabetes, albuminuria, and parathyroid hormone (PTH) (25). By contrast, Seiler et al. investigated the Klotho level in 321 patients with stage 2–4 CKD, in which the Klotho level did not significantly differ based on the CKD stage, and was not associated with eGFR according to Spearman correlation analysis (26).

Previous studies did not focus on the association of Klotho and CKD per se in patients with MetS. Given these knowledge gaps, we leveraged baseline cross-sectional data obtained from National Health and Nutrition Examination Survey (NHANES), to validate the association between Klotho and CKD in patients with MetS and the optimal levels of Klotho, thus providing more precise information regarding Klotho for kidney health and healthy aging.

## 2 Materials and methods

### 2.1 Data sources

The National Center for Health Statistics (NCHS) conducts the National Health and Nutrition Examination Survey (NHANES), a multistage probability sample of the civilian, noninstitutionalized US population (27). NHANES gathers a range of health-related information, such as demographic traits, physical examination results, lab findings, and dietary behavior. All participants provided written informed consent, and the NHANES study protocols received approval from the National Center for Health Statistics (NCHS) Research Ethics Review Board. Detailed information can be accessed at www.cdc.gov/nchs/nhanes/.

### 2.2 Study design and population

**Figure 1:**
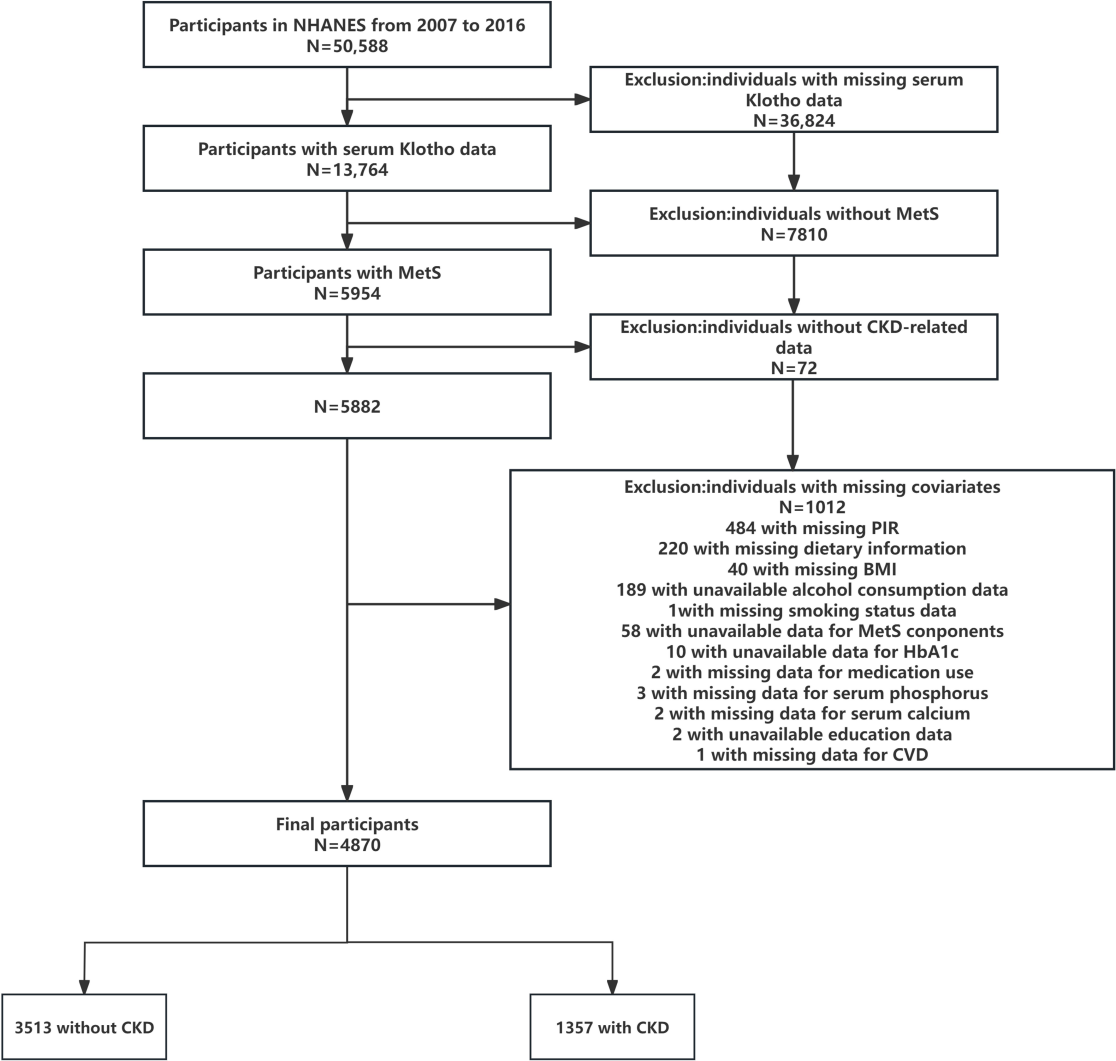
Flow diagram of the screening and enrollment of study participants. MetS, metabolic syndrome; CKD, chronic kidney disease; PIR, family poverty income ratio; BMI, body mass index; CVD, cardiovascular disease.

Data from five successive NHANES cycles conducted between 2007 and 2016 were combined for this cross-sectional analysis. In the NHANES investigation, serum Klotho levels were only assessed in adults between the ages of 40 and 79 (the youngest participant in this sample was 40 years old) (28). Among the 50,588 participants aged 40–79 years, the exclusion criteria were as follows: (a) participants with unavailable data on serum Klotho (n = 36,824); (b) participants without MetS (n = 7810); (c)participants without CKD-related data (n=72); and (c) incomplete covariates(n=1012). Ultimately, 4870 eligible participants were included in the final analysis, as shown in **Figure 1**.

### 2.3 Determination of serum Klotho levels

Serum samples collected from NHANES 2007–2016 participants aged 40–79 years, who consented to the use of their samples for future research, were analyzed with a commercially available ELISA kit (Immuno-Biological Laboratories Co., Ltd., Fujioka, Japan) (29). All samples were kept at -80°C prior to experiments. Duplicate analyzes of samples were conducted in accordance with the manufacturer’s protocol, and all the results were scrutinized against the laboratory’s predefined criteria for acceptability before being finalized for reporting. Samples with duplicate results surpassing a 10% discrepancy were identified for reanalysis. If a quality control sample’s value deviated beyond 2 standard deviations from the expected value, the entire analytical run was rejected, necessitating a repetition of sample analyzes (30). A more detailed description of the laboratory methodology can be found at https://wwwn.cdc.gov/Nchs/Nhanes/2015-2016/SSKL_I.htm.

### 2.4 Definition of MetS

The definition of Metabolic Syndrome (MetS) followed the revised National Cholesterol Education Program Adult Treatment Panel (NCEP-ATP) III criteria, requiring the presence of three or more of the following components (3): (1) elevated waist circumference (WC) defined as a waist circumference of 102 cm for men and 88 cm for women; (2) elevated blood pressure defined as a blood pressure of 130/85 mmHg or medication treatment of previously diagnosed hypertension; (3) reduced high-density lipoprotein cholesterol (HDL-C) defined as an HDL level of<40 mg/dL for men and<50 mg/dL for women or medication treatment for reduced HDL-C; (4) elevated triglyceride (TG) defined as TG level ≥eve mg/dL or medication treatment for elevated TG; and (5) elevated fasting glucose defined as a fasting glucose level of ≥fve mg/L or medication treatment for elevated glucose and previously diagnosed type 2 diabetes.

### 2.5 Assessment of CKD

The estimated glomerular filtration rate (eGFR) was calculated using the Chronic Kidney Disease Epidemiology Collaboration (CKD-EPI) equation (31), which takes into account sex, age, and serum creatinine (Scr) levels. The formula used for calculation is:


$$eGFR=141\times min{\left(Scr/\kappa ,\;1\right)}^{a}\times max{\left(Scr/\kappa ,\;1\right)}^{-1.209}\times 0.993^{Age}\times 1.018\left[iffemale\right]\times 1.159\left[if\;black\right]$$

where *Scr* represents serum creatinine, *κ* = 0.7 (females) or 0.9 (males), *α* is -0.329 for females and -0.411 for males, min indicates the minimum of *Scr/κ* or 1, and max indicates the maximum of *Scr/κ* or 1 (31).

Urine samples from NHANES participants were collected at a standardized mobile examination center (MEC). A fluorescent immunoassay was utilized to quantify the albumin content of the urine. The Jaffé rate reaction, which forms a red creatinine-picrate complex when creatinine and picrate react in an alkaline solution, is used in the urine creatinine analysis (32). A modified Jaffé kinetic technique and solid-phase fluorescence immunoassay were used to measure the levels of creatinine and albumin in the urine. The urinary albumin-to-creatinine ratio (UACR) was calculated by dividing the urinary albumin concentration in milligrams by the urinary creatinine concentration in grams. An eGFR< 60 mL/min/1.73 m^2^ and/or a UACR ≥ 30 mg/g were used to diagnose CKD.

### 2.6 Assessment of covariates

Standardized questionnaires obtained information on sociodemographic characteristics; smoking status, alcohol consumption; physical activity; dietary intake; presence of hypertension, hyperlipidemia, CVD and diabetes; medication use; and biochemical profiles. The participants were divided into groups according to self-reported races and ethnicities: non-Hispanic white, non-Hispanic black, Mexican-American, and other groups (including multiracial participants because NHANES did not provide a detailed list of all races and ethnicities in this category). The family poverty income ratio (PIR) was categorized as ≤1.0, 1–3, or >3.0. Educational background was categorized as below high school (≤ 9th grade or 9–11th grade including 12th grade without diploma), high school or equivalent, and above high school (some college or associate’s degree or college graduate or above).

Prior to the interview at the mobile examination center (MEC), participants underwent a dietary recall interview to gather their 24-hour nutritional data, encompassing total dietary intake of calories, protein, carbohydrates, fat, and fiber. Body mass index (BMI), waist circumference (WC), and various biochemical indicators were derived from medical evaluations and laboratory tests conducted at the MEC. BMI was divided into three categories with cutoff values of 25 and 30 kg/m^2^, corresponding to “normal<25, overweight 25–30, or obesity ≥30”, respectively.

In terms of smoking status, individuals who reported smoking fewer than 100 cigarettes throughout their lifetime were categorized as never smokers. Those who had smoked more than 100 cigarettes and were currently smoking were identified as current smokers, whereas former smokers were individuals who had smoked over 100 cigarettes but had stopped smoking (33).

The criteria used to diagnose alcohol consumption and status were as follows: current heavy drinking, defined as consuming a minimum of 4 drinks per day for men, 3 drinks per day for women, or engaging in binge drinking on 5 or more days per month. Current moderate drinking was characterized by consuming at least 3 drinks per day for men, 2 drinks per day for women, or binge drinking on 2 or more days per month. Individuals who did not meet these criteria were classified as current light drinkers. Additionally, individuals were categorized as never drinkers if they had consumed fewer than 12 drinks in their lifetime, and as former drinkers if they had consumed 12 or more drinks in a year but did not drink in the last year, or if they did not drink in the last year but had consumed 12 or more drinks in their lifetime (33).

Histories of medical conditions were self-reported. CVD was determined by a combination of self-reported physician diagnoses and standardized medical status questionnaires completed during individual interviews. DM was defined as (1) doctor diagnosed diabetes; (2) glycohemoglobin >6.5%; (3) fasting glucose ≥7.0 mmol/L; (4) random blood glucose ≥11.1 mmol/L; (5) 2-h oral glucose tolerance test blood glucose ≥11.1 mmol/L; and (6) use of diabetes medication or insulin (34). Glycosylated hemoglobin A1c (HbA1c) measurements were performed by the Diabetes Laboratory at the University of Minnesota using a Tosoh A1c 2.2 Plus Glycohemoglobin Analyzers (Tosoh Medics, Inc., San Francisco, CA). All blood pressure readings were obtained during a single visit. An average of up to three consecutive systolic and diastolic blood pressure readings were used. Hypertension was diagnosed if the participant meets any of the following: (1) systolic blood pressure ≥ 140 mmHg or diastolic blood pressure ≥ 90 mmHg was found greater than or equal to three times during the physical examination; (2) previously diagnosed with hypertension; and (3) on medication for hypertension (35). In the meantime, if any of the three criteria were met by the participants, hyperlipidemia was diagnosed: triglycerides (TG) ≥ 1.69 mmol/L, total cholesterol (TC) ≥ 5.17 mmol/L, or low-density lipoprotein (LDL) ≥ 3.36 mmol/L; or high-density lipoprotein (HDL)< 1.03 mmol/L (male)/1.29 mmol/L (female); or were prescribed lipid-lowering drugs (36). Data concerning medications consumed in the last 30 days were gathered by skilled professionals who matched the products supplied by participants with a database containing information on drugs and dietary supplements.

In addition, strict laboratory analyzes were performed, including the assessment of serum calcium and serum phosphorus at baseline. The full measurement technique for these variables is available at https://www.cdc.gov/nchs/nhanes/. Details of the laboratory analyzes are documented in the NHANES Laboratory Medical Technologists Procedures Manual.

### 2.7 Statistical analysis

The survey procedure was adapted to the complex survey design and appropriate sample weights were applied for this random subsample to be representative of the civilian population of the United States, as recommended by the National Center for Health Statistics (NCHS). Normally distributed variables were presented as mean ± standard deviations (SDs), while skewed variables were presented as median (interquartile range). Categorical variables were presented in the form of numbers and percentages. To test differences across quartiles of serum Klotho levels, one-way analysis of variance (ANOVA) and Kruskal-Wallis tests were conducted for continuous variables with normal and skewed distributions, respectively. Pearson’s chi-square tests were performed for categorical variables. Serum Klotho concentrations were log base 2 transformed due to its skewed distribution, and it is also rational to explain that the change of the dependent variable is caused by per doubling of the exposure.

Odds ratios (ORs) and 95% confidence intervals (95% CIs) were calculated to analyze the association between Klotho and CKD using survey-weighted logistic regression models. Participants were divided into four groups based on log2-transformed Klotho quartile amounts: Q1 (7.24–9.32 pg/mL; n = 1218), Q2 (9.33–9.63 pg/mL; n = 1217), Q3 (9.64–9.94 pg/mL; n = 1217), Q4 (9.95–12.30 pg/mL; n = 1218), and the lowest quartile served as the reference group. We filter covariates based on the following four criteria: (1) demographic data; (2) factors affecting CKD and Klotho levels reported in the literature (18, 25, 28, 29, 37); (3) changes in regression coefficients of the basic model exceeding 10% (38); and (4) our clinical experience. As an effort for reducing methodological bias, we also used the directed acyclic graph (DAG) approach. The DAG provides a graphical representation of the variables related to the exposure and the outcome and is helpful in identifying potential sources of bias. We used the DAGitty software, version 3.1, to make our DAG, presented in **Supplementary Figure S1**, suggesting that minimal sufficient adjustment sets for estimating the total effect of serum Klotho on CKD: components of MetS and the number of Mets components, lifestyle factors (waist circumference, alcohol drinking, physical activity, and smoking), medication use, pre-existing diseases (CVD, diabetes, hyperlipidemia, hypertension), serum phosphorus and calcium, and socialdemographics (age, sex, race, PIR, and education).

Unadjusted, minimally adjusted, and fully adjusted findings were presented in line with the STROBE guidelines: Model 1, a non-adjusted crude model. Model 2: adjusted for social demographic characteristics (age, sex, and race). Model 3: adjusted for Model 2 plus covariates based on factors that changed the matched OR by at least 10% when it was added to this model, including HbA1c, CVD, anti-hypertensive medication use, and lipid-lowering medication use. Model 4: adjusted for Model 3 plus the covariate P<0.1 in univariate model, comorbidities, and other variables reported in the previous literature, including education, PIR, waist circumference, smoking status, alcohol drinking status, protein intake, fiber intake, fat intake, carbohydrate, diabetes, hypertension, hyperlipidemia, anti-diabetic medication use, physical activity, serum calcium, serum phosphorus, components of MetS, and the number of MetS components. We used Klotho as both continuous and categorical variables and calculated the *P*_trend_, used to verify the result of Klotho as a continuous variable.

To validate the correlation between Klotho and CKD and to explore the possibility of a nonlinear relationship between Klotho and CKD, a weighted generalized additive model (GAM) was also used to visually assess the relationship between the two (39), which was selected for its flexibility in modeling nonlinear relationships without assuming a specific functional form. This approach is particularly suitable for large datasets like NHANES, as it can detect complex patterns while maintaining statistical power. For nonlinear correlations, a segmented regression model was established to calculate the relationship between Klotho and CKD using a smoothing plot. Log-likelihood ratio test comparing one-line (non-segmented) model with segmented regression (also known as piecewise regression) model was also applied to assess the robustness of the results. When the relationship between Klotho and CKD was apparent in the smoothed curve, the method automatically calculated the inflection point by maximizing the model likelihood.

To ascertain if the association between serum Klotho protein and CKD incidence was consistent across several subgroups and to identify potential effect modifiers, we performed interaction and stratified analyzes by age (40–60 years; 61–79 years), sex (male; female), BMI (<25; 25–30; >30), diabetes status (no; pre diabetes; yes), hypertension status (no; yes), smoking status (never; former; now), race (white; black; Mexican American; other) and five MetS components (yes; no). In each subgroup, we examined the association between Klotho concentration and the odds of CKD. Subgroup analyzes were performed using stratified linear regression models, while modifications and interactions among subgroups were examined through likelihood ratio tests.

To assess the robustness of our findings, we also performed several sensitivity analyzes. First, calculations were repeated with the Modification of Diet in Renal Disease study (MDRD) eGFR equation (40). Second, the International Diabetes Federation (IDF) 2009 was used as the diagnostic criteria for MetS to see if the results were consistent (41). Third, the parameter estimates may be biased as a result of the analysis of the missing data of 1,012 participants. Subsequently, we implemented multiple imputation to maintain the participants in the analysis. Fourth, to yield comparable groups across different Klotho levels, we performed propensity score matching (PSM). Propensity scores were calculated with a logistic regression where Klotho levels (divided by median) was the dependent variable. The aforementioned factors were chosen as covariates in order to produce the propensity score. Individuals in the two groups were matched 1:1 (42). The estimated propensity scores were then utilized as weighting factors. Pairwise algorithmic (PA) (43), standardized mortality ratio weight (SMRW) (44) models were used to generate a weighted cohort to adjust the baseline confounders, thus reflecting more truly the independent association between Klotho levels and CKD. We also explored the potential for unmeasured confounding between Klotho and CKD by calculating E-values (45).

Given that the sample size was determined exclusively using the provided data, no prior statistical power assessments were carried out (46). All data analysis and visualization were conducted using R software (version 4.3.1 http://www.R-project.org, The R Foundation), EmpowerStats software (http://www.empowerstats.com, X&Y solutions, Inc., Boston, MA, USA) and the Free Statistics software (version 1.9.1; Beijing FreeClinical Medical Technology Co., Ltd, Beijing, China). *P*<0.05 (two-sided) was considered statistically significant.

## 3 Results

### 3.1 Baseline characteristics of participants

Clinical and biochemical characteristics of participants according to the Klotho quartiles are summarized in **Table 1**. A total of 4870 participants selected from NHANES 2007–2016 were involved in our study, representing approximately 38,872,250 US adults aged 40–79 years. Based on the weighted analyzes, 51.6% were females, with an average age of 57.7 ± 0.2 years. The median (interquartile range, IQR) of serum Klotho was 782.9 (641.3, 968.2) pg/mL. A total of 1357 participants (22.4%) were classified as having CKD. The mean baseline eGFR was 84.5 ± 0.3 ml/min/1.73 m^2^. The prevalence of CKD was higher among participants with lower Klotho levels (Q1: 420[28.1%]) than among participants with higher Klotho levels (Q3: 292[18.7%]). Across quartiles of Klotho, differences with statistical significance were observed regarding age; race; drinking status; smoking status; BMI; eGFR; CVD; CKD; daily energy, carbohydrate, fiber, fat, and protein intake level (all *P*< 0.05).

**Table 1:** Basic characteristics of NHANES participants (N = 4870).

	Total	Klotho levels quartiles (pg/mL)				*P* Value
Characteristics^*^	(n = 4870)	Q1(n = 1218)	Q2(n = 1217)	Q3(n = 1217)	Q4(n = 1217)	
Age, years**^†^**	57.7±0.2	59.0±0.4	57.4±0.4	57.6±0.4	56.9±0.4	0.001
Female, n (%)^‡^	2584(51.6)	627(52.3)	605(48.4)	660(51.4)	692(54.6)	0.220
Education, n (%)						0.473
Below high school	1411(17.3)	359(17.0)	347(16.9)	363(18.0)	342(17.5)	
High school	1195(25.6)	329(28.1)	278(23.4)	312(26.6)	276(24.3)	
Above high school	2264(57.1)	530(55.0)	592(59.7)	542(55.5)	600(58.2)	
PIR, n (%)						0.607
≤ 1.0	1014(12.0)	255(11.5)	246(11.0)	254(12.5)	259(13.2)	
1–3	2144(36.7)	557(37.6)	527(38.5)	542(36.0)	518(34.5)	
> 3.0	1712(51.3)	406(50.9)	444(50.5)	421(51.4)	441(52.4)	
Race, n (%)						< 0.001
White	2295(75.8)	601(76.8)	605(77.8)	596(76.7)	493(71.7)	
Black	855(7.9)	222(8.4)	188(6.6)	173(6.1)	272(10.9)	
Mexican American	863(7.0)	201(6.0)	208(6.4)	230(7.7)	224(8.1)	
Other	857(9.2)	194(8.9)	216(9.2)	218(9.5)	229(9.3)	
Drinking status, n (%)						0.010
Former	1250(22.7)	330(23.4)	297(20.9)	306(21.6)	317(25.2)	
Never	774(11.3)	165(9.8)	190(11.1)	200(11.7)	219(13.0)	
Mild	1573(37.6)	371(35.2)	403(38.8)	407(38.3)	392(38.1)	
Moderate	587(14.1)	147(15.5)	145(12.4)	136(14.2)	159(14.5)	
Heavy	686(14.3)	205(16.1)	182(16.9)	168(14.3)	131(9.3)	
Smoking status, n (%)						0.056
Former	1604(34.0)	425(35.3)	420(35.0)	405(34.7)	354(30.8)	
Never	2353(47.8)	528(44.7)	565(45.3)	594(48.8)	666(52.9)	
Now	913(18.2)	265(20.0)	232(19.7)	218(16.5)	198(16.3)	
Physical activity, n (%)						0.120
Inactive	1845(42.1)	428(42.9)	503(45.6)	465(40.9)	449(38.6)	
Active	3025(57.9)	790(57.1)	714(54.4)	752(59.1)	769(61.4)	
Physical examination						
BMI, kg/m^2^	32.9±6.3	32.5±6.1	32.9±5.9	33.2±6.5	33.1±6.7	0.022
WC, cm	111.0±0.3	110.3±0.4	111.3±0.4	111.5±0.5	110.8±0.7	0.205
Laboratory data						
eGFR, ml/min/1.73 m^2^	84.5±0.3	80.0±0.7	85.0±0.6	85.7±0.6	87.7±0.6	<0.001
UACR, mg/g^§^	9.0 [5.5, 21.3]	9.0 [5.4, 23.2]	9.0 [5.5, 21.4]	8.5 [5.5, 19.6]	9.5 [5.6, 21.8]	0.259
HbA1c, %	6.2±1.2	6.0±1.0	6.1±1.1	6.1±1.1	6.4±1.6	< 0.001
Phosphorus, mmol/L	1.2±0.2	1.2±0.0	1.2±0.0	1.2±0.0	1.2±0.0	0.420
Calcium, mmol/L	2.4±0.0	2.3±0.0	2.4±0.0	2.3±0.0	2.4±0.0	0.108
Dietary intake						
Energy, kcal/d	2079.0±19.6	2014.4±35.2	2157.7±34.9	2079.0±42.2	2058.0±32.6	0.033
Carbohydrate, g/d	244.1±2.5	233.9±4.1	250.7±4.5	244.6±5.3	247.0±4.4	0.022
Dietary fiber, g/d	16.6±0.2	15.8±0.4	17.5±0.4	16.6±0.4	16.5±0.4	0.043
Fat intake, g/d	82.5±1.1	78.8±1.8	85.8±1.8	83.5±2.0	81.7±1.7	0.038
Protein intake, g/d	81.2±0.8	77.0±1.4	84.4±1.6	81.1±1.6	82.1±1.5	0.002
Medication use, n (%)						
Glucose-lowering drugs	1520(25.5)	412(26.7)	359(25.4)	365(23.8)	384(26.1)	0.642
Antihypertensive drugs	3252(63.3)	865(67.8)	808(62.5)	783(59.5)	796(63.5)	0.068
Lipid-lowering drugs	2049(39.9)	566(43.6)	515(40.6)	499(38.0)	469(37.3)	0.286
Comorbidities, n (%)						
Hypertension	3552(69.8)	921(72.4)	889(68.7)	864(68.2)	878(70.1)	0.316
Diabetes	2177(37.0)	542(36.0)	504(34.9)	529(36.1)	602(41.5)	0.172
CVD	957(16.9)	300(20.5)	227(14.8)	222(16.7)	208(15.8)	0.026
Hyperlipidemia	4566(93.9)	1151(94.6)	1144(94.7)	1148(93.7)	1123(92.6)	0.391
Events, n (%)						
CKD	1357(22.4)	420(28.1)	336(21.9)	292(18.7)	309(20.8)	< 0.001

^*^All estimates accounted for complex survey designs.^†^Normally distributed continuous variables are described as means ± SEs.^‡^Categorical variables are presented as numbers (percentages). ^§^Continuous variables without a normal distribution are described as medians (interquartile ranges).

OR, odds ratio; CI, confidence interval; BMI, body mass index; WC, waist circumference; PIR, family poverty income ratio; eGFR, estimated glomerular filtration rate; UACR, urine albumin-creatinine ratio; HbA1c, glycosylated hemoglobin A1c; HDL, high-density lipoprotein; CVD, cardiovascular disease; CKD, chronic kidney disease;SE, standard error.

**Figure 2:**
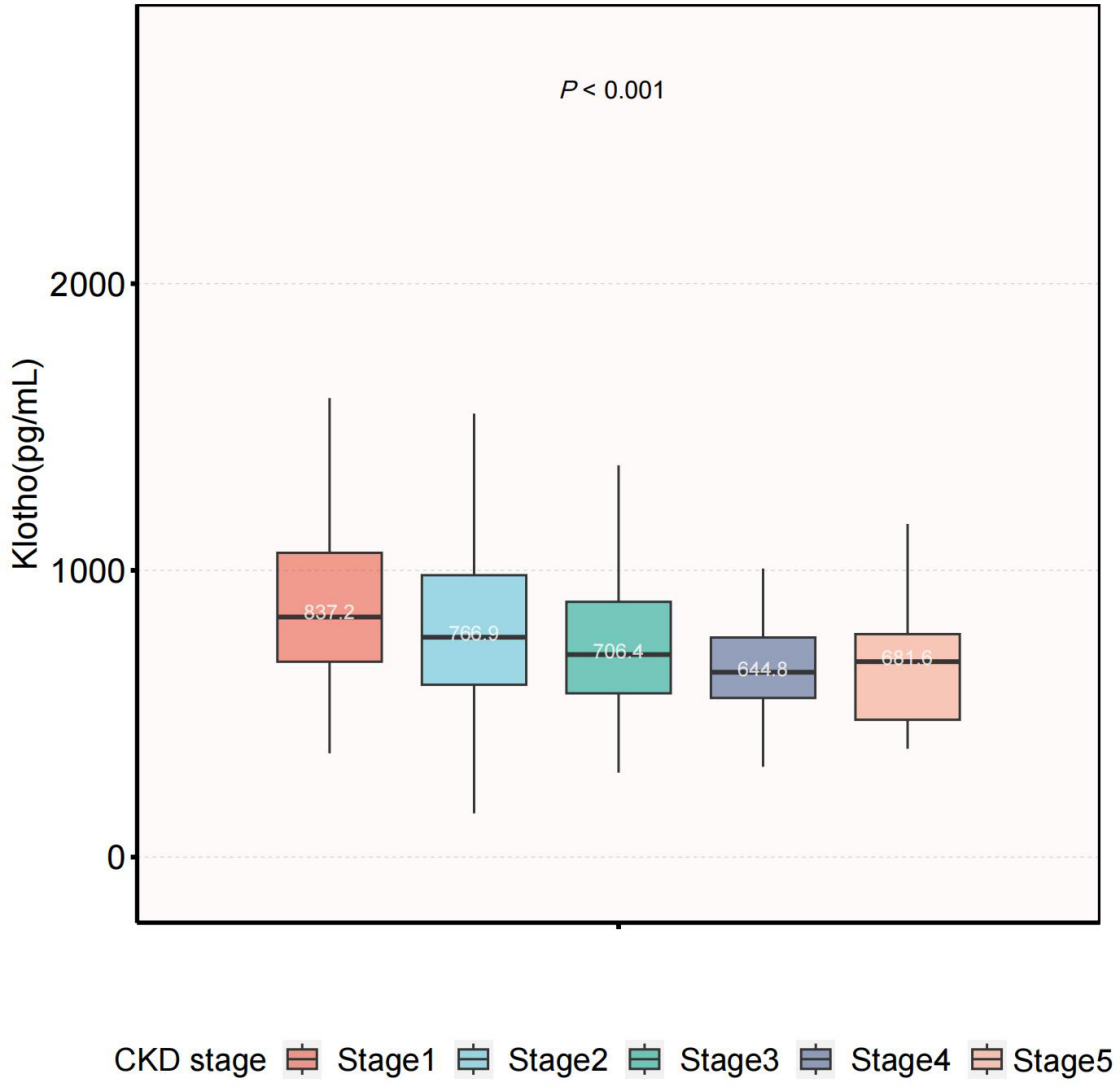
Serum Klotho levels across CKD stages. The level of serum Klotho decreased with more advanced stage (P<0.001). CKD, chronic kidney disease.

In our study, participants with higher Klotho levels were likely to be female and had hypertension. Serum Klotho levels across CKD stages are shown in **Figure 2**. Significant differences were observed among the five groups (*P<*0.001), and the median value of Klotho in the CKD stage 4 was the lowest.

### 3.2 Association between serum Klotho levels and the risk of CKD

**Figure 3:**
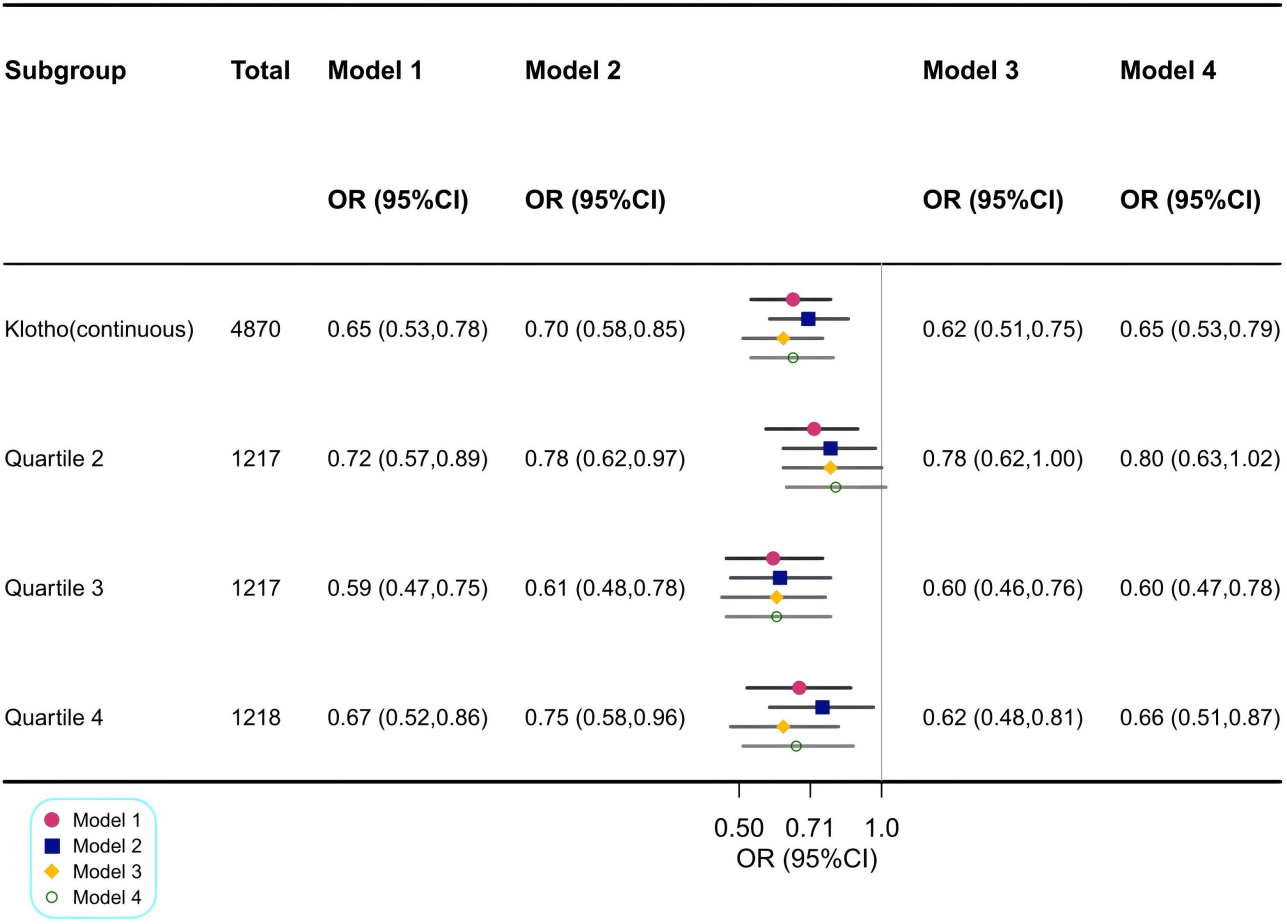
Association between log2-transformed Klotho and CKD. Model 1: Crude model. Model 2: Adjusted for age, sex and race. Model 3: Adjusted for Model 2 + HbA1c, CVD, anti-hypertensive medication use and lipid-lowering medication use. Model 4: Adjusted for Model 3 + education, PIR, waist circumference, smoking status, alcohol drinking status, protein intake, fiber intake, fat intake, carabohydrate, diabetes, hypertension, hyperlipidemia, anti-diabetic medication use, physical activity, serum calcium, serum phosphorus, components of MetS and the number of MetS components. OR, odds ratio; CI, confidence interval; CKD, chronic kidney disease; HbA1c, glycosylated hemoglobin A1c; CVD, cardiovascular disease; PIR, family poverty income ratio; MetS, metabolic syndrome; FBG, fasting blood glucose.

The association between serum Klotho levels and the prevalence of CKD is detailed in **Figure 3**. Our results suggested that higher Klotho levels were significantly associated with decreased risk of CKD both in the crude model [odds ratio (OR), 0.65; 95% CI, 0.53–0.78] and the minimally adjusted Model 2 (OR, 0.70; 95% CI, 0.58–0.85). In the fully adjusted Model 4, the negative association between Klotho and CKD remained stable (OR, 0.65; 95% CI, 0.53–0.79), indicating that when log2-transformed Klotho concentrations increased one unit, the odds of CKD were 35% lower.

Similar findings were observed when Klotho was included in the regression model as a categorical variable, divided into four quartiles, with the lowest log2-transformed Klotho level (first quartile) as the reference. In fully adjusted Model 4, the decreases in serum Klotho levels for the second quartile, third quartile, and fourth quartile were 20% (95% CI, 0.63–1.02), 40% (95% CI, 0.47–0.78), and 34% (95% CI, 0.51–0.87), respectively.

**Figure 4:**
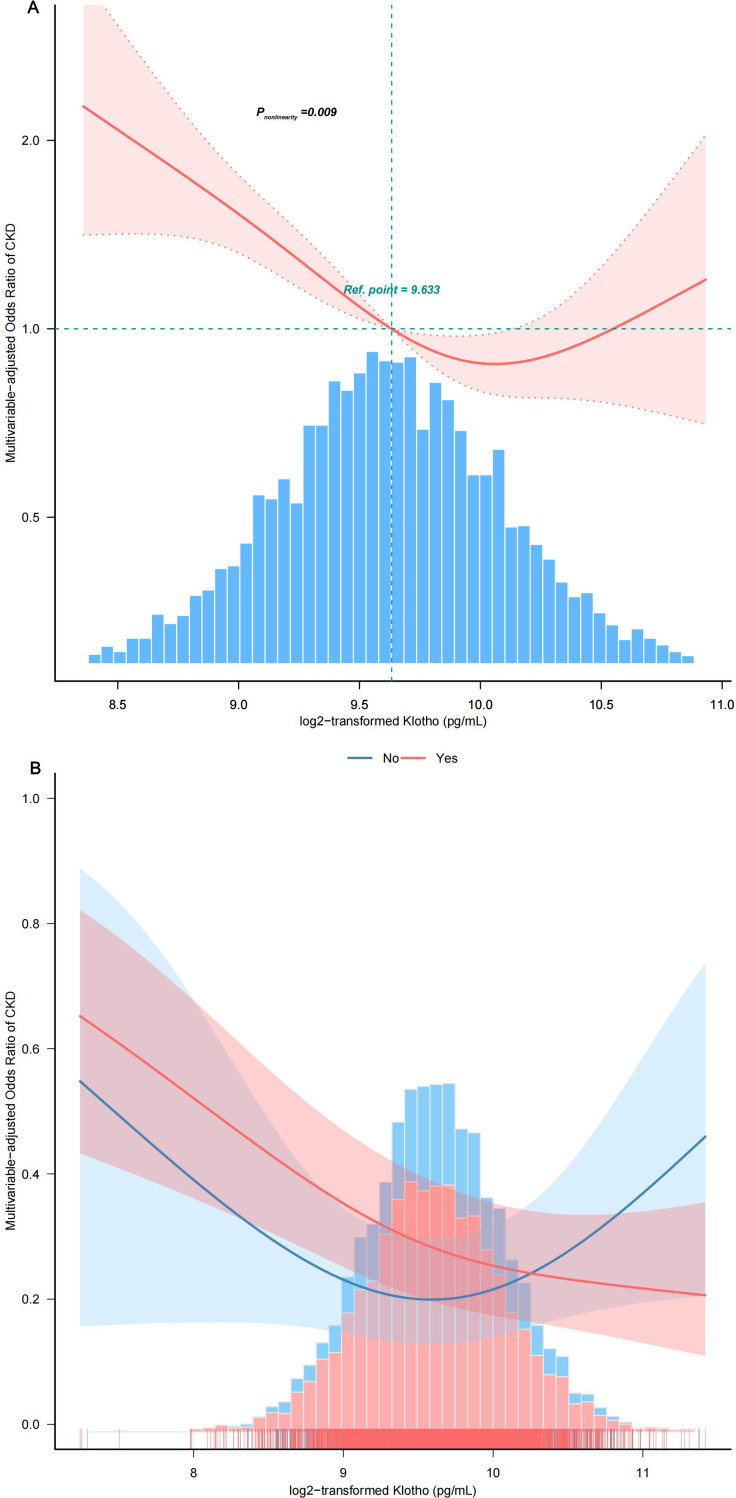
Smooth curve fitting. **(A)** Association between log2-transformed Klotho and the incidence of CKD. **(B)** Association between Klotho log2 transform and the incidence of CKD stratified by hypertension status. The solid and dashed lines represent the odds ratios and their corresponding 95% confidence intervals. Only 99% of the data is displayed after adjusting variables in Model 4. PIR, family poverty income ratio; HbA1c, glycosylated hemoglobin A1c; CVD, cardiovascular disease; MetS, metabolic syndrome; FBG, fasting blood glucose.

By utilizing a two-piecewise logistic regression model within a generalized additive model (GAM), the inflection point at 9.88 pg/mL was identified. Accordingly, the association between Klotho and CKD exhibited an L-shaped curve (*P*_nonlinearity_ =0.009) in spline curve fitting (**Figure 4A**). In the threshold analysis, the OR of developing CKD was 0.57 (95%CI, 0.47–0.70) in participants with a Klotho level of 9.88 pg/mL (**Table 2**), which means that the risk of CKD is reduced by 43% with every one unit increase in serum Klotho concentration. Serum Klotho levels were not associated with CKD when Klotho was >9.88 pg/mL (OR,1.11; 95% CI, 0.77–1.60), indicating that the risk of CKD no longer decreased with increasing serum Klotho concentrations.

**Table 2:** Threshold effect analysis of Klotho on CKD using the two-piecewise logistic regression model.

Models	CKD§	
	OR (95%CI)	*P* value
Model 1^*^		
One line effect	0.69 (0.60, 0.80)	<0.001
Model 2^†^		
Turning point(K)	9.88	
<9.88 slope1	0.57 (0.47, 0.70)	<0.001
>9.88 slope2	1.11 (0.77, 1.60)	0.586
slope2-1	1.94 (1.19, 3.14)	0.008
Model fit value at K	-1.19 (-1.30, -1.08)	
Log-likelihood ratio test^‡^	–	0.008

^*^Model 1: linear analysis. ^†^Model 2: nonlinear analysis. ^‡^Log-likelihood ratio test: *P* value<0.05 means Model 2 is significantly different from Model 1, which indicates a nonlinear relationship. Only 99% of the data is displayed. ^§^Adjusted for variables in Model 4.

CKD, chronic kidney disease; OR, odds ratio; CI, confidence interval.

### 3.3 Subgroup analysis

**Figure 5:**
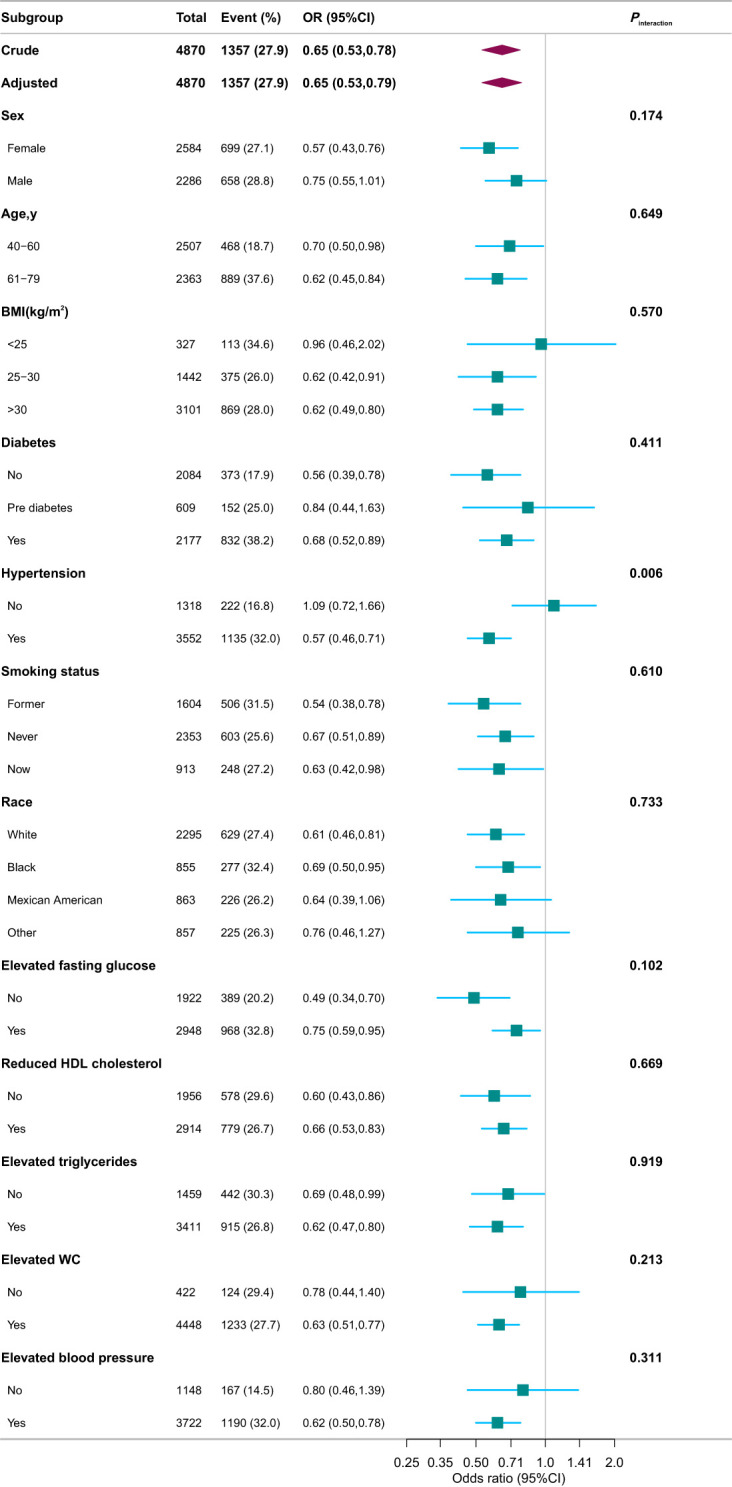
Associations between serum Klotho and CKD in different subgroups. Except for the stratification component itself, each stratification factor was adjusted for variables in Model 4. OR, odds ratio; CI, confidence interval; PIR, family poverty income ratio; HbA1c, glycosylated hemoglobin A1c; CVD, cardiovascular disease; HDL, high-density lipoprotein; WC, waist circumference.

Stratified and interaction analyses were performed to investigate potential modifications in the relationship between serum Klotho protein levels and the prevalence of CKD across various subgroups (**Figure 5**). After adjusting for potential confounders, consistent findings were noted across strata defined by age, sex, race, PIR, educational attainment, physical activity, smoking habits, alcohol consumption, components of MetS, BMI, and diabetes status. Additionally, hypertension status produced an intriguing significant interaction whereby in patients with hypertension, every 1-pg/mL increase in log2 Klotho was associated with a 43% decrease in CKD risk, while in individuals with no hypertension, every 1-pg/mL increase in log2 Klotho was associated with a 9% increase in CKD risk (*P*_interaction_ =0.006) (**Figure 4B**).

### 3.4 Sensitivity analysis

To support our conclusions, we conducted sensitivity analyzes. The results supported the main results described above. Largely similar results were seen in sensitivity analyzes when the MDRD equation was used instead to define CKD (**Supplementary Table S1**). In fully adjusted model, the association was negative between Klotho and CKD (OR,0.74; 95% CI, 0.61–0.90). In addition, when MetS was redefined by IDF2009, after the adjustment of all covariates in Model 4, the association between Klotho and CKD remained (OR, 0.62; 95% CI, 0.51–0.77) (**Supplementary Table S2**). Multiple imputation analysis yielded consistent results (**Supplementary Table S3**). The results remained stable even after accounting for possible confounders using PSM and controlling for propensity score, pairwise algorithmic, overlap weight, and doubly robust analysis. The ORs were 0.64–0.76, and *P* values were all<0.001(**Supplementary Figure S2**). For the current study, the OR at serum Klotho concentrations for risks of CKD was 0.65 with a 95% CI of 0.53-0.79 (shown in **Figure 2**). The E-value for this point estimate is 2.45. Thus, following the suggested language of VanderWeele and Ding (47), the correlation between serum Klotho and the risk of CKD was found to be robust, unless the OR of the risk of CKD of an unmeasured confounder was >2.45.

### 3.5 Summary of results regarding Klotho concentration and CKD risk

Our analysis reveals a significant L-shaped relationship between serum Klotho levels and CKD risk in individuals with metabolic syndrome. A specific Klotho concentration range (9.63 to 9.94 pg/mL) is linked to the lowest prevalence of CKD. Notably, a critical inflection point at 9.88 pg/mL demonstrates that lower serum Klotho levels are associated with increased CKD risk, especially in hypertensive patients. This threshold suggests that maintaining Klotho levels within this optimal range may be essential for reducing CKD risk in individuals with MetS. Our findings highlight Klotho’s potential clinical significance as a biomarker for kidney health and intervention strategies.

## 4 Discussion

In this nationally representative cross-sectional study involving 4,870 patients with metabolic syndrome (MetS) from the 2007–2016 NHANES, we found a significant independent negative association between serum Klotho levels and the risk of chronic kidney disease (CKD), indicating a 35% reduction in risk. Our findings revealed a dose-response relationship described by an L-shaped curve (P_nonlinearity_ = 0.009), suggesting that the association is most pronounced at Klotho levels below 9.88 pg/mL. This threshold underscores the importance of maintaining Klotho levels within this optimal range to mitigate CKD risk, particularly in hypertensive patients (P_interaction_ = 0.006).

Our results are consistent with previous studies indicating that systemic Klotho levels decrease in CKD patients and decline further as estimated glomerular filtration rate (eGFR) declines. For example, Drew et al. identified an independent association between higher soluble Klotho levels and a reduced risk of kidney function decline in individuals without albuminuria (25). However, their study’s small sample size limits its generalizability. Similarly, Zhang et al. reported a positive relationship between Klotho levels and eGFR and a negative association with advanced CKD stages (37), but did not specifically analyze individuals with MetS, who are particularly vulnerable to renal abnormalities.

Some observational studies yield inconsistent results. Seiler et al. (26) and Bob et al. (48) reported no significant association between Klotho levels and kidney function, with variations attributed to differences in study populations, CKD stages, and methodologies. Discrepancies may stem from complex factors such as oxidative stress, inflammation, medications (49, 50), and disease progression that influence Klotho levels. The fluctuating nature of Klotho concentrations, especially in advanced CKD stages and during dialysis (13, 51, 52), further complicates these observations.

Our study provides new insights into the nonlinear relationship between Klotho levels and CKD risk, with a critical inflection point at 9.88 pg/mL. This finding implies that Klotho’s renoprotective effects are most pronounced at higher levels, whereas CKD risk sharply increases below this threshold. The mechanisms underlying this relationship may involve Klotho’s roles in regulating mineral metabolism (53), inhibiting fibrosis (54), and modulating oxidative stress (21) and inflammation (54). Additionally, Klotho deficiency has been associated with the activation of pro-inflammatory and pro-fibrotic pathways (55, 56), which are critical drivers of CKD progression. This identified inflection point may represent a threshold where these detrimental pathways predominate, leading to rapid deterioration in kidney function. At optimal levels, Klotho may effectively maintain these homeostatic processes, but below a critical threshold, protective mechanisms may be impaired, resulting in increased CKD risk. These findings emphasize the importance of Klotho management in clinical practice, particularly for patients with MetS, who are at heightened risk for CKD (3).

The interaction with hypertension further complicates Klotho’s role in kidney function. Our results suggest that Klotho deficiency may create a vicious cycle with hypertension, potentially increasing CKD risk through mechanisms such as impaired endothelial function (24, 57) and activation of inflammatory pathways (55, 56).

The identified threshold of 9.88 pg/mL serves as a potential clinical target for Klotho management. Clinicians should consider monitoring Klotho levels as an early indicator of kidney health, particularly in patients with MetS and hypertension. Our study has several strengths, including a large sample size that ensures robust statistical power, representation of the general U.S. population, which enhances external validity, and rigorous adherence to standardized data collection methods to minimize non-sampling and measurement errors according to stringent NHANES criteria. Additionally, complex sampling weights and sample design were applied to achieve a nationally representative sample estimate.

While our study presents compelling evidence, it is essential to acknowledge its limitations. First, the cross-sectional design of our study precludes establishing causality between CKD and MetS. While a strong correlation is evident, the directionality of the association and potential confounding factors remain unclear. This design does not capture temporal changes in metabolic parameters, which could oversimplify the CKD-MetS relationship. Reverse causality is also a possibility, as lifestyle modifications in CKD patients might influence metabolic parameters. Future research should focus on longitudinal studies and randomized controlled trials to enhance causal inference.

Second, serum creatinine and albuminuria were assessed only once, limiting our ability to evaluate dynamic changes in serum Klotho concentrations. While baseline measurements are informative, fluctuations due to various influences may affect the validity of our findings. Consequently, our results should be considered preliminary, emphasizing the need for longitudinal studies with multiple assessments to further investigate Klotho’s role in renal function.

Third, our findings are based on middle-aged and older adults with MetS in the U.S., which may restrict generalizability. Additionally, though the ELISA kit used for Klotho measurement underwent stringent quality control (46), precision concerns persist. Nonetheless, our large sample size and advanced statistical methodologies help mitigate the effects of batch-to-batch variation. Future studies should utilize multiple measurement techniques for Klotho to improve accuracy.

Lastly, unmeasured confounding variables could impact the relationship between α-Klotho and CKD. We were unable to account for serum fibroblast growth factor 23 (FGF23), which plays a critical role in phosphate homeostasis and kidney function (58, 59). Further multicenter studies are necessary to clarify this relationship. While we adjusted for related minerals in our regression model, other factors such as nutritional status (28) and oxidative stress biomarkers (55) may still influence associations. The observed odds ratio of 0.65 for incident CKD could be impacted by an unmeasured confounder with a risk ratio exceeding 1.79. However, it is improbable that such confounding would negate the effect of serum Klotho levels, as this ratio exceeds those for established CKD risk factors. Given the consistent findings across various models, the observed relationships are unlikely to be attributable to random chance.

Future studies should prioritize longitudinal research to establish causal links between Klotho levels and CKD progression, investigate the molecular mechanisms behind the observed L-shaped relationship, and develop targeted interventions to maintain Klotho levels within the optimal range, particularly for patients with MetS and hypertension. Additionally, exploring the impact of Klotho management on CKD progression in diverse populations could enhance our understanding of its clinical implications.

## 5 Conclusion

An L-shaped association was found between serum Klotho levels and the risk of CKD in patients with MetS, with an inflection point at 9.88 pg/mL. The findings from our study consistently indicated that both extremely low and high Klotho levels were linked to a heightened risk of CKD. This underscores the significance of regulating serum Klotho levels within an optimal range to prevent CKD in middle-aged and older individuals with MetS.

## Data Availability

Publicly available datasets were analyzed in this study. This data can be found here: https://wwwn.cdc.gov/nchs/nhanes.
