# Supplementary material for: Nonlinear relationship between serum Klotho and chronic kidney disease in US adults with metabolic syndrome
Source: Front Endocrinol (Lausanne). 2024 Dec 24;15:1409560. doi: 10.3389/fendo.2024.1409560 (PMC11703749; doi:10.3389/fendo.2024.1409560)
Supplement: Supplementary file 1 [file DataSheet1.docx]

Supplementary Material

| Supplementary Table 1. Association between serum soluble Klotho and CKD calculated with MDRD equation.  \|  \| N \| Model 1^*^ \| \| Model 2^†^ \| \| Model 3^‡^ \| \| Model 4^§^ \| \| \| --- \| --- \| --- \| --- \| --- \| --- \| --- \| --- \| --- \| --- \| \| OR (95%CI) \| *P* value \| OR (95%CI) \| *P* value \| OR (95%CI) \| *P* value \| OR (95%CI) \| *P* value \| \| Klotho \| 4870 \| 0.74 (0.62,0.88) \| <0.001 \| 0.80 (0.67,0.95) \| .010 \| 0.76 (0.64,0.92) \| .004 \| 0.74 (0.61,0.90) \| .004 \| \| Quartile1 \| 1218 \| Ref \| - \| Ref \| - \| Ref \| - \| Ref \| - \| \| Quartile2 \| 1217 \| 0.77 (0.62,0.96) \| .020 \| 0.84 (0.67,1.06) \| .13 \| 0.85 (0.67,1.07) \| .17 \| 0.85 (0.67, 1.08) \| .17 \| \| Quartile3 \| 1217 \| 0.64 (0.52,0.79) \| <0.001 \| 0.66 (0.54,0.82) \| <0.001 \| 0.67 (0.54,0.83) \| <0.001 \| 0.67 (0.54, 0.84) \| <0.001 \| \| Quartile4 \| 1218 \| 0.74 (0.59,0.93) \| .010 \| 0.81 (0.65,1.03) \| .08 \| 0.76 (0.60,0.96) \| .020 \| 0.73 (0.57, 0.94) \| .020 \| \| *P*_trend_ \| - \| - \| .002 \| - \| .010 \| - \| .003 \| - \| .003 \|   ^*^Model 1: Crude model  ^†^Model 2: Adjusted for age, sex and race.  ^‡^Model 3: Adjusted for model 2 + HbA1c, CVD, anti-hypertensive medication use and lipid-lowering medication use.  ^§^Model 4: Adjusted for model 3 + education, PIR, waist circumference, smoking status, alcohol drinking status, protein intake, fiber intake, fat intake, carbohydrate, diabetes, hypertension, hyperlipidemia, anti-diabetic medication use, physical activity, serum calcium, serum phosphorus, components of MetS and the number of MetS components.  The results did not significantly change when we used the MDRD equation instead to define CKD.  **Abbreviation:** OR, odds ratio; CI, confidence interval; CKD, chronic kidney disease; HbA1c, glycosylated hemoglobin A1c; CVD, cardiovascular disease; PIR, family poverty income ratio; MetS, metabolic syndrome; FBG, fasting blood glucose. Supplementary Table 2. Association between serum soluble Klotho and CKD redefined with IDF2009.  \|  \| N \| Model 1^*^ \| \| Model 2^†^ \| \| Model 3^‡^ \| \| Model 4^§^ \| \| \| --- \| --- \| --- \| --- \| --- \| --- \| --- \| --- \| --- \| --- \| \| OR (95%CI) \| *P* value \| OR (95%CI) \| *P* value \| OR (95%CI) \| *P* value \| OR (95%CI) \| *P* value \| \| Klotho \| 4870 \| 0.63(0.52,0.76) \| <0.001 \| 0.68(0.57,0.82) \| <0.001 \| 0.59(0.48,0.72) \| <0.001 \| 0.62(0.51,0.77) \| <0.001 \| \| Quartile1 \| 1218 \| Ref \| - \| Ref \| - \| Ref \| - \| Ref \| - \| \| Quartile2 \| 1217 \| 0.70(0.56,0.88) \| 0.003 \| 0.75(0.59,0.96) \| 0.020 \| 0.74(0.58,0.96) \| 0.020 \| 0.73(0.57,0.94) \| 0.010 \| \| Quartile3 \| 1217 \| 0.54(0.44,0.68) \| <0.001 \| 0.56(0.45,0.70) \| <0.001 \| 0.53(0.42,0.67) \| <0.001 \| 0.55(0.43,0.70) \| <0.001 \| \| Quartile4 \| 1218 \| 0.60(0.47,0.77) \| <0.001 \| 0.67(0.53,0.85) \| 0.001 \| 0.53(0.41,0.69) \| <0.001 \| 0.57(0.43,0.75) \| <0.001 \| \| *P*_trend_ \| - \| - \| <0.001 \| - \| <0.001 \| - \| <0.001 \| - \| <0.001 \|   ^*^Model 1: Crude model.  ^†^Model 2: Adjusted for age, sex and race.  ^‡^Model 3: Adjusted for model 2 + HbA1c, CVD, anti-hypertensive medication use and lipid-lowering medication use.  ^§^Model 4: Adjusted for model 3 + education, PIR, waist circumference, smoking status, alcohol drinking status, protein intake, fiber intake, fat intake, carbohydrate, diabetes, hypertension, hyperlipidemia, anti-diabetic medication use, physical activity, serum calcium, serum phosphorus, components of MetS and the number of MetS components.  Consistent results were demonstrated when MetS was redefined by IDF2009 (see the fully adjusted model 4).  **Abbreviation:** OR, odds ratio; CI, confidence interval; CKD, chronic kidney disease; HbA1c, glycosylated hemoglobin A1c; CVD, cardiovascular disease; PIR, family poverty income ratio; MetS, metabolic syndrome; FBG, fasting blood glucose. |
| --- | --- | --- | --- | --- | --- | --- | --- | --- | --- | --- | --- | --- | --- | --- | --- | --- | --- | --- | --- | --- | --- | --- | --- | --- | --- | --- | --- | --- | --- | --- | --- | --- | --- | --- | --- | --- | --- | --- | --- | --- | --- | --- | --- | --- | --- | --- | --- | --- | --- | --- | --- | --- | --- | --- | --- | --- | --- | --- | --- | --- | --- | --- | --- | --- | --- | --- | --- | --- | --- | --- | --- | --- | --- | --- | --- | --- | --- | --- | --- | --- | --- | --- | --- | --- | --- | --- | --- | --- | --- | --- | --- | --- | --- | --- | --- | --- | --- | --- | --- | --- | --- | --- | --- | --- | --- | --- | --- | --- | --- | --- | --- | --- | --- | --- | --- | --- | --- | --- | --- | --- | --- | --- | --- | --- | --- | --- | --- | --- | --- | --- | --- | --- | --- | --- | --- | --- | --- | --- | --- | --- | --- | --- | --- | --- | --- | --- | --- | --- | --- | --- | --- | --- | --- | --- | --- | --- |

# Supplementary Table 3. Association between serum Klotho and CKD using complete case analysis and multiply imputed data.

|  | CKD (n=1357) | | | |
| --- | --- | --- | --- | --- |
|  | ^*^Number with missing data | | ^*^Complete cases | |
|  | OR (95% CI) | *P* value | OR (95% CI) | *P* value |
| Klotho (continuous) | 0.69 (0.6~0.8) | <0.001 | 0.7 (0.62~0.8) | <0.001 |
| Q1 | Ref | - | Ref | - |
| Q2 | 0.81 (0.67~0.98) | 0.026 | 0.82 (0.69~0.97) | 0.021 |
| Q3 | 0.63 (0.52~0.76) | <0.001 | 0.63 (0.53~0.75) | <0.001 |
| Q4 | 0.64 (0.53~0.78) | <0.001 | 0.65 (0.55~0.77) | <0.001 |
| *P*_trend_ |  | <0.001 |  | <0.001 |
| ^*^Adjusted covariates: age, sex, race, education, PIR, waist circumference, HbA1c, smoking status, alcohol drinking status, protein intake, fibre intake, fat intake, carbohydrate, diabetes, CVD, hypertension, hyperlipidemia, anti-diabetic medication use, anti-hypertensive medication use, lipid-lowering medication use, physical activity, serum calcium, serum phosphorus, components of MetS and the number of MetS components.  The results were generally robust when using a multiple imputation to address missing data.  **Abbreviation:** OR, odds ratio; CI, confidence interval; CKD, chronic kidney disease; HbA1c, glycosylated hemoglobin A1c; CVD, cardiovascular disease; PIR, family poverty income ratio; MetS, metabolic syndrome; FBG, fasting blood glucose. | | | | |

# **Supplementary Figure S1.** Directed acyclic graph (DAG) explaining the association between the exposures, the outcome, and covariates included in the analyses.


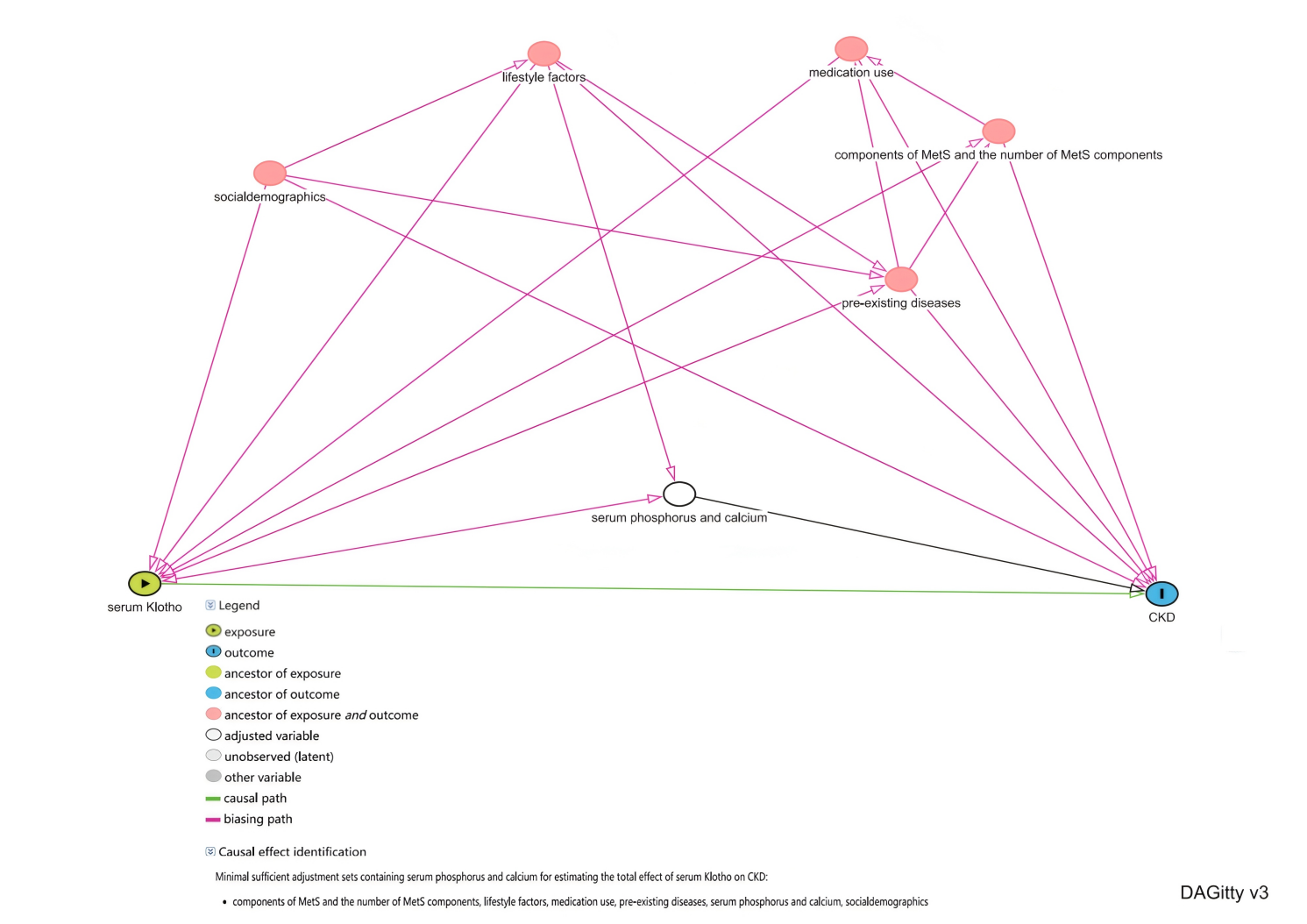


DAG suggests minimal sufficient adjustment sets for estimating the total effect of serum Klotho on CKD: components of MetS and the number of Mets components, lifestyle factors (waist circumference, alcohol drinking, physical activity, and smoking), medication use, pre-existing diseases (CVD, diabetes, hyperlipidemia, hypertension), serum phosphorus and calcium, and socialdemographics (age, sex, race, PIR, and education).

DAG was drawn using http://www.dagitty.net/

# **Supplementary Figure S2.** Primary outcome analysis with five different models: (1) multivariable logistic regression model, (2) propensity score adjusted model, (3) propensity score matching model, (4) propensity score PA model, (5) propensity score IPTW model, (6) doubly robust model with all covariates.


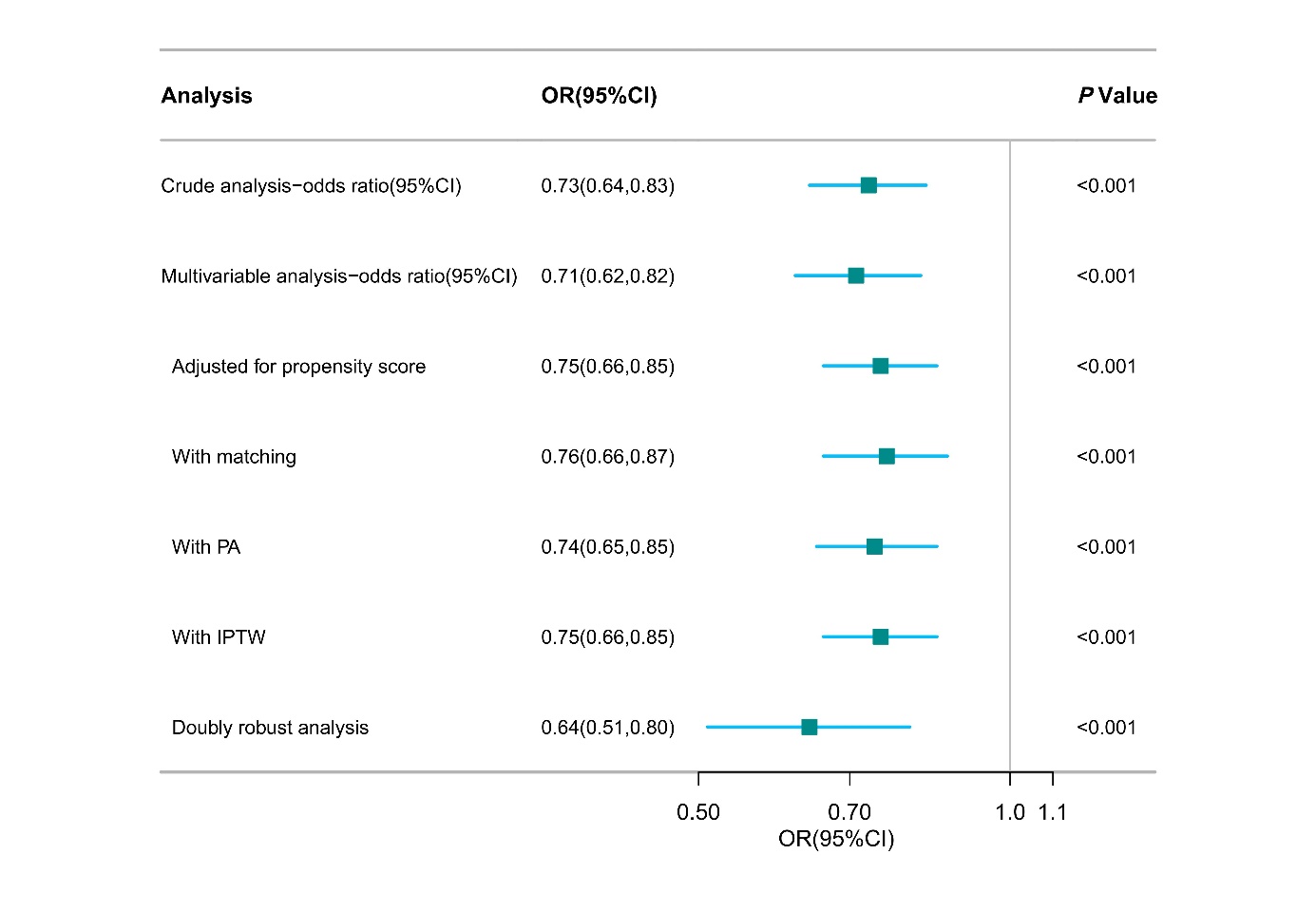


Propensity scores were calculated with the use of a logistic regression model including all the covariates in model 4. A significantly lower risk of CKD was found in univariate, multivariable logistic regression analyses, PSM, adjusted for propensity score, PA, IPTW, and doubly robust estimate.

**Abbreviation:** OR, odds ratio; CI, confidence interval; PSM, propensity score matching; PA, pairwise algorithmic; IPTW, inverse probability of treatment weighting.
